# Selective Inhibition of Matrix Metalloproteinase-9 Attenuates Secondary Damage Resulting from Severe Traumatic Brain Injury

**DOI:** 10.1371/journal.pone.0076904

**Published:** 2013-10-23

**Authors:** Orr Hadass, Brittany N. Tomlinson, Major Gooyit, Shanyan Chen, Justin J. Purdy, Jennifer M. Walker, Chunyang Zhang, Andrew B. Giritharan, Whitley Purnell, Christopher R. Robinson, Dmitriy Shin, Valerie A. Schroeder, Mark A. Suckow, Agnes Simonyi, Grace Y. Sun, Shahriar Mobashery, Jiankun Cui, Mayland Chang, Zezong Gu

**Affiliations:** 1 Department of Pathology and Anatomical Sciences, University of Missouri School of Medicine, Columbia, Missouri, United States of America; 2 Department of Biochemistry, University of Missouri School of Medicine, Columbia, Missouri, United States of America; 3 Center for Translational Neuroscience, University of Missouri School of Medicine, Columbia, Missouri, United States of America; 4 MS in Pathology Program, University of Missouri Graduate School, Columbia, Missouri, United States of America; 5 Interdisciplinary Neuroscience Program, University of Missouri Graduate School, Columbia, Missouri, United States of America; 6 Department of Psychological Sciences, University of Missouri, Columbia, Missouri, United States of America; 7 Department of Chemistry and Biochemistry, University of Notre Dame, Notre Dame, Indiana, United States of America; 8 Freimann Life Sciences Center and Department of Biological Sciences, University of Notre Dame, Notre Dame, Indiana, United States of America; University of Portsmouth, School of Pharmacy & Biomedical Sciences, United Kingdom

## Abstract

Traumatic brain injury (TBI) is a leading cause of death and long-term disability. Following the initial insult, severe TBI progresses to a secondary injury phase associated with biochemical and cellular changes. The secondary injury is thought to be responsible for the development of many of the neurological deficits observed after TBI and also provides a window of opportunity for therapeutic intervention. Matrix metalloproteinase-9 (MMP-9 or gelatinase B) expression is elevated in neurological diseases and its activation is an important factor in detrimental outcomes including excitotoxicity, mitochondrial dysfunction and apoptosis, and increases in inflammatory responses and astrogliosis. In this study, we used an experimental mouse model of TBI to examine the role of MMP-9 and the therapeutic potential of SB-3CT, a mechanism-based gelatinase selective inhibitor, in ameliorating the secondary injury. We observed that activation of MMP-9 occurred within one day following TBI, and remained elevated for 7 days after the initial insult. SB-3CT effectively attenuated MMP-9 activity, reduced brain lesion volumes and prevented neuronal loss and dendritic degeneration. Pharmacokinetic studies revealed that SB-3CT and its active metabolite, *p*-OH SB-3CT, were rapidly absorbed and distributed to the brain. Moreover, SB-3CT treatment mitigated microglial activation and astrogliosis after TBI. Importantly, SB-3CT treatment improved long-term neurobehavioral outcomes, including sensorimotor function, and hippocampus-associated spatial learning and memory. These results demonstrate that MMP-9 is a key target for therapy to attenuate secondary injury cascades and that this class of mechanism-based gelatinase inhibitor–with such desirable pharmacokinetic properties–holds considerable promise as a potential pharmacological treatment of TBI.

## Introduction

Traumatic brain injury (TBI) is a devastating condition that results in significant morbidity and mortality and affects over a million individuals in the United States each year. Survivors of this type of severe brain trauma can experience lifelong disabilities. TBI resulting from a primary penetrating or non-penetrating injury to the head is frequently caused by falls, motor vehicle accidents, sports injuries, and firearm incidents [1]. After the initial insult, secondary injury develops within hours to days and even weeks. Biochemical, metabolic and cellular changes observed during the secondary phase are typically associated with disruption of the blood-brain barrier (BBB), inflammatory responses and infiltration of blood-derived macrophages, edema, and cell death [2]. Secondary brain injury is thought to be responsible for the development of many of the sustained neurological deficits after TBI. Their delayed onset may also provide a window of opportunity for therapeutic intervention. There is substantial evidence of the important role of matrix metalloproteinases (MMPs) associated with injury-induced neurovascular impairment and remodeling during secondary brain damage [3], [4].

MMPs are members of a family of 26 zinc-dependent endopeptidases that have structurally similar hemopexin, propeptide, and catalytic domains [5]. They regulate homeostasis of the extracellular matrix (ECM) by proteolysis of its components, such as collagen, laminin, and fibronectin [6]. Pathological activation of MMPs, in particular MMP-9, has been shown to cause a number of detrimental outcomes, including BBB disruption, hemorrhage, neuronal apoptosis [7], and brain damage in ischemic stroke [8] and TBI [3], [9], [10]. Although the mechanisms for activation of MMP-9 remain elusive, there is evidence for involvement of free radicals, such as nitric oxide, in triggering its activation [8], [11]. The critical role of MMP-9 in the pathology of TBI is also supported by recent clinical studies, in which elevated levels of MMP-9 were observed in ventricular cerebrospinal fluid in patients with severe TBI [12]. These results suggest that MMP-9 could be a key target for therapy to attenuate several of the secondary injury cascades responsible for brain damage after a TBI.

Current understanding of secondary injury mechanisms points to the need for pleiotropic neuroprotective agents that can target multiple biochemical pathways. However, attempts to develop neuroprotective drugs to alleviate secondary damage resulting from TBI in humans have been confronted with challenges, due largely to inadequate understanding of the biochemical mechanisms underlying secondary injury, lack of therapeutic concentrations in the brain, and insufficient testing of drugs during the clinical therapeutic window that can mitigate the progression of brain damage [2]. The fact that more than 98 percent of small-molecule drugs do not penetrate into the brain has been a major roadblock in the development of effective therapeutics for neurodegenerative diseases [13].

SB-3CT, the first mechanism-based MMP inhibitor developed by our research team, is known to target gelatinases (MMP-2 and MMP-9) with high selectivity due to a unique mechanism of inhibition that is catalyzed by the target gelatinases themselves [14]. We first reported that this mechanism-base inhibitor exhibits profound neuroprotection in mouse models of cerebral ischemia [15], [16]. Administration of SB-3CT attenuates ECM proteolysis, rescues neurons and reduces hemorrhage. To shed light on the efficacy of SB-3CT on neuroprotection, we investigated the metabolism of SB-3CT [17], [18]. SB-3CT is metabolized primarily by hydroxylation at the *para* position of the terminal phenyl ring (*p*-OH SB-3CT) and this derivative is a more potent gelatinase inhibitor compared with the parent compound.

Although SB-3CT has been recognized as a benchmark selective gelatinase inhibitor in various studies [9], [19], [20], its efficacy in mitigating the secondary damage of TBI has not been investigated. In the present study, we used the electromagnetic (EM) impactor to precisely induce a controlled cortical impact (CCI) in mice, as a model of TBI [21]. This study describes the cellular response to the injury and the resulting behavior deficits, as well as the role of MMP-9 in mediating TBI-induced brain damage. We further examine the efficacy of SB-3CT in mitigating damage to the brain, and ameliorating behavioral deficits on sensorimotor and cognitive functions. In addition, the ability of SB-3CT and its metabolite to distribute to the brain was investigated, which is as a pre-requisite for the ultimate marketing approval of any drug.

## Results

### Time Course of MMP Gelatinolytic Activity after TBI

**Figure 1:**
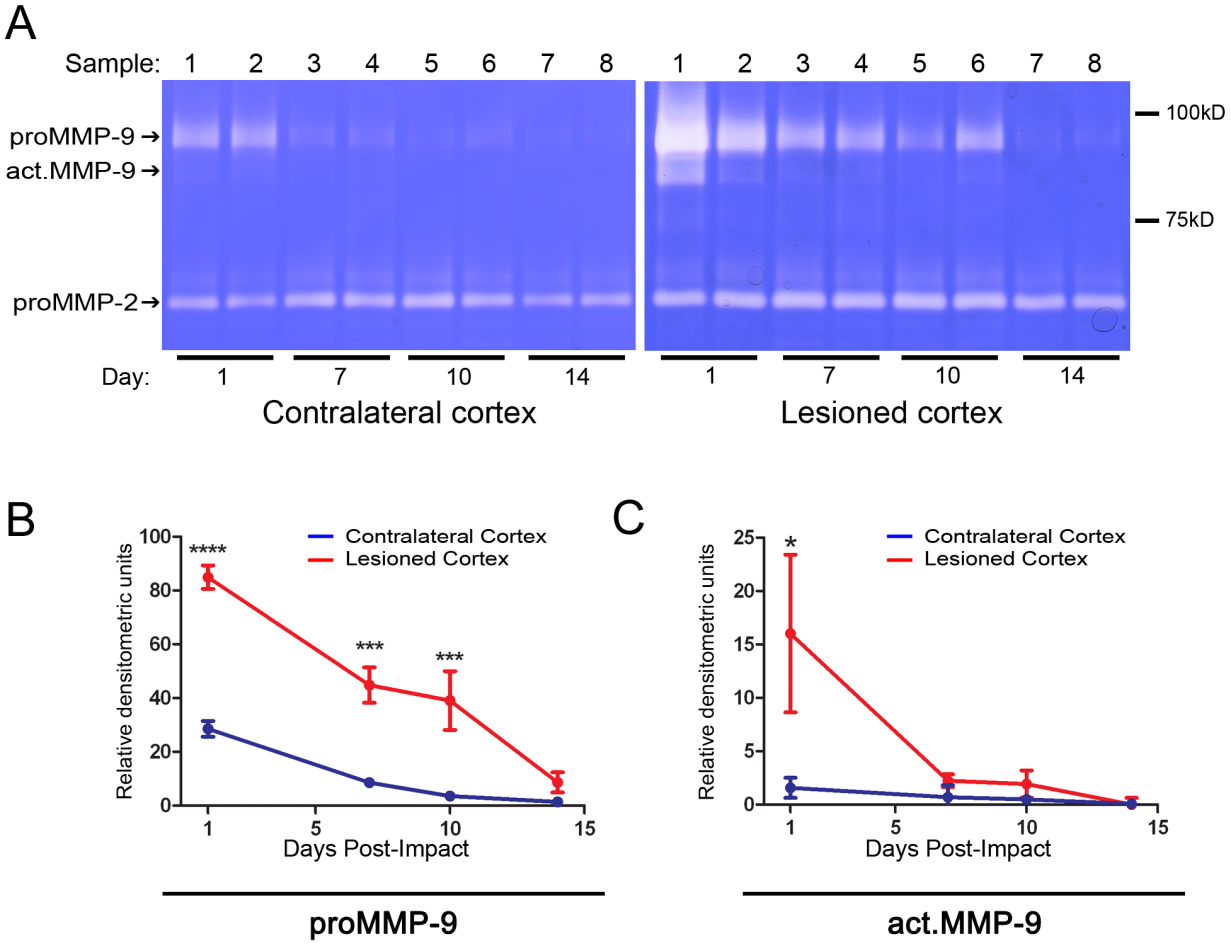
Time course of MMP gelatinolytic activity after TBI in mice. ***A.*** Representative zymograms of mouse cortical tissue at different time points post-trauma. Gelatin zymography showed gelatinolytic bands representing the latent (proMMP-9) and activated form (act.MMP-9) of MMP-9 in different time courses. ***B*** and ***C.*** Densitometry analysis of gelatinolytic bands shown in (***A***) representing the proMMP-9 (***B***) and act.MMP-9 (***C***). n = 5 at each time point; *, **, ***, and ****, *p*<0.05, 0.01, 0.001, and 0.0001, respectively, by one-way ANOVA using Dunnett’s multiple comparison test; data are expressed as means ± SEM.

Previous studies have reported that MMP activity in the central nervous system (CNS) is induced by different types of injuries [3], [9], [22]. In the present study, the EM impactor-induced TBI mouse model was used to characterize the time course of gelatinolytic activity as measured by gelatin zymography. Representative zymograms are shown for 1, 7, 10, and 14 days post-trauma (Figure 1A). Densitometric analysis of the gelatinolytic activity showed that both the inactive latent MMP-9 (proMMP-9) and active MMP-9 levels in the lesioned cortex were elevated 24 hours after trauma. This was followed by a decline at 7 and 10 days in both proMMP-9 and active MMP-9 (Figures 1B and 1C). At 14 days after trauma, active MMP-9 was not detected. We also noted a small transient increase in proMMP-9 in the contralateral cortex at 24 hours after TBI (Figures 1A and 1B) similar to a previous report [22]. Under these experimental conditions, proMMP-2 levels did not change over the two-week period study (Figure 1A). Our findings are consistent with previous reports that MMP-2 is constitutively expressed, whereas MMP-9 is inducible in rodents [9], [15], [16] and in humans [23]–[25] after brain injuries.

### SB-3CT Treatment Reduces MMP-9 Activity in the Mouse Brain after TBI

**Figure 2:**
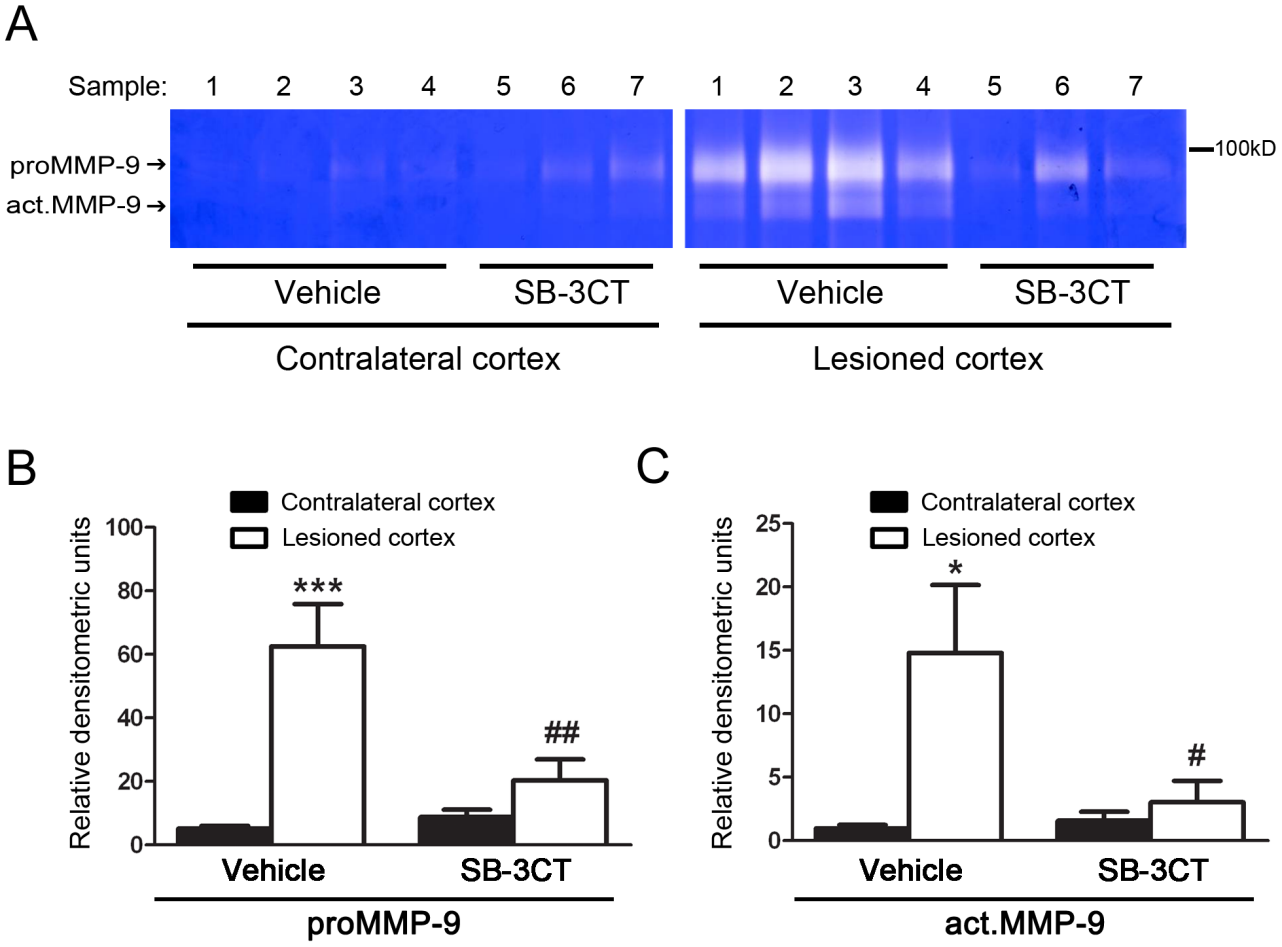
Attenuation of MMP-9 gelatinolytic activity after SB-3CT treatment. ***A.*** Representative zymograms comparing cortical MMP-9 levels in SB-3CT versus vehicle-treated mice at 7 days post-trauma. Gelatin zymography revealed increased levels of proMMP-9 and act.MMP-9 in the lesioned cortex of vehicle-treated mice, whereas in SB-3CT-treated mice that activity was significantly attenuated. ***B***–***C.*** Densitometry measurements of proMMP-9 (***B***) and act.MMP-9 (***C***) at 7 days post-trauma. n = 7 in vehicle-treated, and 6 in SB-3CT-treated mice; * and ***, *p*<0.05 and 0.001, respectively, comparing lesioned to contralateral cortex in vehicle-treated mice; ^#^ and ^##^, p<0.05 and 0.01, respectively, comparing the difference between the contralateral and lesioned cortex after SB-3CT treatment to that in vehicle-treated mice by one-tailed, unpaired Student’s *t*-test; data are expressed as means ± SEM.

To examine whether SB-3CT can attenuate aberrant MMP-9 activity after TBI, this compound was administered intraperitoneally (i.p.) at 25 mg/kg/day for 7 days. The dose and duration of the treatment were based on our previous publication showing protective effects of SB-3CT in ischemic stroke [16]. We dosed for 7 days, as gelatin zymography revealed increased active MMP-9 in the lesioned cortex for 7 days after TBI. SB-3CT treatment attenuated both the levels of proMMP-9 and active MMP-9 (Figure 2A). Densitometry confirmed that SB-3CT treatment lowered the levels of both proMMP-9 (Figure 2B) and active MMP-9 (Figure 2C).

### SB-3CT and its Active Metabolite, *p*-OH SB-3CT, Cross the BBB

**Figure 3:**
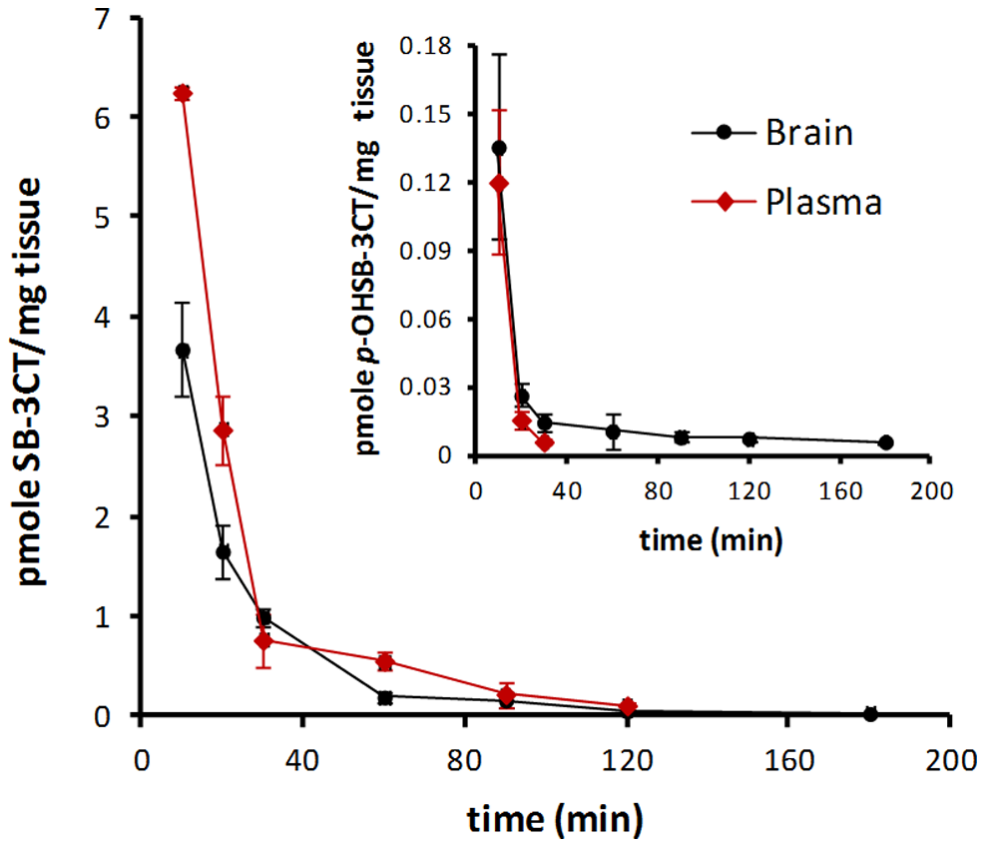
Brain and plasma concentrations versus time curves of SB-3CT and *p*-OH SB-3CT after repeated 25 mg/kg i.p. administration of SB-3CT to mice. Samples were analyzed by reversed-phase UPLC/ESI with MRM. SB-3CT and its active metabolite *p*-OH SB-3CT were rapidly absorbed and distributed to the brain. Brain levels of SB-3CT were above its *K_i_* for MMP-9, while those of *p*-OH SB-3CT were below its *K_i_* for MMP-9, suggesting that the efficacy seen in the TBI model is probably due to the ability of the parent SB-3CT to cross the BBB.

To investigate whether the mechanism-based MMP inhibitors could cross the BBB, we recently developed and validated a highly sensitive bioanalytical method based on ultraperformance liquid chromatography (UPLC) using electrospray ionization (ESI) in the negative ion mode with multiple reactive monitoring (MRM) detection to measure levels of SB-3CT and its active metabolite, *p*-OH SB-3CT, in plasma and brain [17]. Using this method, we evaluated the ability of SB-3CT and its active metabolite, *p*-OH SB-3CT, to cross the BBB following repeated i.p. administration of SB-3CT at 25 mg/kg to healthy mice. Brain levels of SB-3CT of 3.7±0.5 pmole/mg were maximal at 10 minutes after the last dose and decreased to 0.022±0.005 pmole/mg at 180 minutes (Figure 3 and Table 1). Levels of SB-3CT in brain remained above the *K_i_* for MMP-9 of 400 nM [26] for 30 minutes. In general, brain levels of SB-3CT were lower than those found in plasma, with plasma *AUC*_0–∞_ (the area under the concentration-time curve from time zero to infinity, which is a measure of the body exposure to a drug) of 134 µM·minutes and brain *AUC*_0–∞_ of 87.8 pmole·minutes/mg (equivalent to 87.8 µM·minutes, assuming a density of 1 g/mL), for a brain to plasma ratio of 0.66. SB-3CT was rapidly absorbed and distributed to the brain, with *t*_½α_ of 8.7 minutes and an elimination half-life *t*_½β_ of 53 minutes. Brain *AUC*_0–∞_ of SB-3CT after administration of three repeated-doses was 87.8 pmole·minutes/mg and was lower than following a single dose (122 pmole·minutes/mg) [17], indicating that SB-3CT did not accumulate in the brain. Likewise, the elimination half-lives after repeated doses and single dose administration of SB-3CT were similar (53 minutes vs. 46 minutes), indicating that SB-3CT was desirably cleared from the brain and did not accumulate.

**Table 1:** Concentrations and pharmacokinetic parameters of SB-3CT and *p*-OH SB-3CT after repeated i.p. administration of SB-3CT to mice.

Time (min)	SB-3CT^a^		*p*-OH SB-3CT^a^	
	Brain	Plasma	Brain	Plasma
**10**	3.7±0.5	6.2±0.1	0.14±0.04	0.12±0.03
**20**	1.6±0.3	2.9±0.3	0.026±0.005	0.016±0.004
**30**	0.99±0.09	0.76±0.26	0.015±0.004	0.0060±0.0010
**60**	0.19±0.05	0.55±0.08	0.011±0.007	NQ^b^
**90**	0.16±0.08	0.22±0.13	0.0081±0.0022	NQ^b^
**120**	0.049±0.013	0.10±0.04	0.0076±0.0009	NQ^b^
**180**	0.022±0.005	NQ^b^	0.0060±0.0004	NQ^b^
***AUC*** **_0–last_** a	86.1	130	2.98	1.45
***AUC*** **_0–∞_** a	87.8	134	4.61	NC^c^
***t*** **_½α_ (min)**	8.7	6.6	4.2	3.4
***t*** **_½β_ (min)**	53	25	187	NC^c^
**Brain** ***_AUC_*** **/Plasma** ***_AUC_***	0.66		2.1	

a Concentrations in µM in plasma and in pmole/mg tissue in brain; *AUC* in µM·minutes in plasma and in pmole·minutes/mg in brain.

b NQ = non-quantifiable.

c NC = not calculated; the low levels observed did not allow for the calculation of the terminal half-life and *AUC*_0–∞**.**_

Levels of *p*-OH SB-3CT after repeated-dose administration of SB-3CT were lower than those of SB-3CT in both plasma and brain (Figure 3 inset and Table 1). Plasma levels were 0.12±0.03 µM 10 minutes after the last dose and were not detected after 60 minutes. Brain levels of *p*-OH SB-3CT were higher than plasma, with values of 0.14±0.04 pmole/mg in brain at 10 minutes, and decreasing to 0.0060±0.0004 pmole/mg at 180 min. Levels of *p*-OH SB-3CT were below its *K_i_* for MMP-9 of 160 nM at all times [18]. Brain *AUC*_0–last_ was 2.98 pmole·minutes/mg and plasma *AUC*_0–last_ was 1.45 µM·minutes; the ratio of brain to plasma *AUC*_0–last_ was 2.1. Hence, *p*-OH SB-3CT was rapidly absorbed and distributed to the brain, with *t*_½α_ of 4.2 minutes and an elimination half-life *t*_½β_ of 187 minutes. Brain *AUC*_0–∞_ for *p*-OH SB-3CT was 4.61 pmole·minutes/mg, while that for SB-3CT was 87.8 pmole·minutes/mg, suggesting that the efficacy seen in the TBI model is likely due to the ability of the parent inhibitor SB-3CT to cross the BBB, rather than SB-3CT being metabolized in the liver to its more potent metabolite *p*-OH SB-3CT and later distributing to the brain.

The regional brain distribution showed the brain region to plasma ratios ranging from 0.39 to 0.60 for SB-3CT and from 0.92 to 1.3 for *p*-OH SB-3CT, indicating that SB-3CT and its metabolite, *p*-OH SB-3CT, were distributed in all regions of the brain (Table 2).

**Table 2:** Regional brain distribution of SB-3CT and *p*-OH SB-3CT after repeated i.p. administration of SB-3CT^b^.

	SB-3CT		*p*-OH SB-3CT	
	Conc^c^	Ratio^d^	Conc^c^	Ratio^d^
**Whole brain**	3.7±0.5	0.60±0.08	0.14±0.04	1.2±0.4
**Brain stem**	2.8±0.8	0.45±0.13	0.13±0.03	1.1±0.4
**Cerebellum**	2.4±0.4	0.39±0.06	0.13±0.03	1.1±0.4
**Cortex**	2.7±0.8	0.44±0.13	0.16±0.05	1.3±0.5
**Hippocampus**	3.1±0.8	0.50±0.13	0.11±0.03	0.92±0.34
**Striatum**	3.5±1.1	0.56±0.18	0.12±0.04	1.0±0.4
**Plasma**	6.2±0.1	–	0.12±0.03	–

*^b^*regional brain distribution determined at 10 minutes after the last i.p. dose of SB-3CT.

*^c^*concentration in pmole/mg tissue and in µM in brain and plasma, respectively.

*^d^*ratio of the concentration in brain region to plasma.

### SB-3CT Treatment Reduces Lesion Volume after TBI

**Figure 4:**
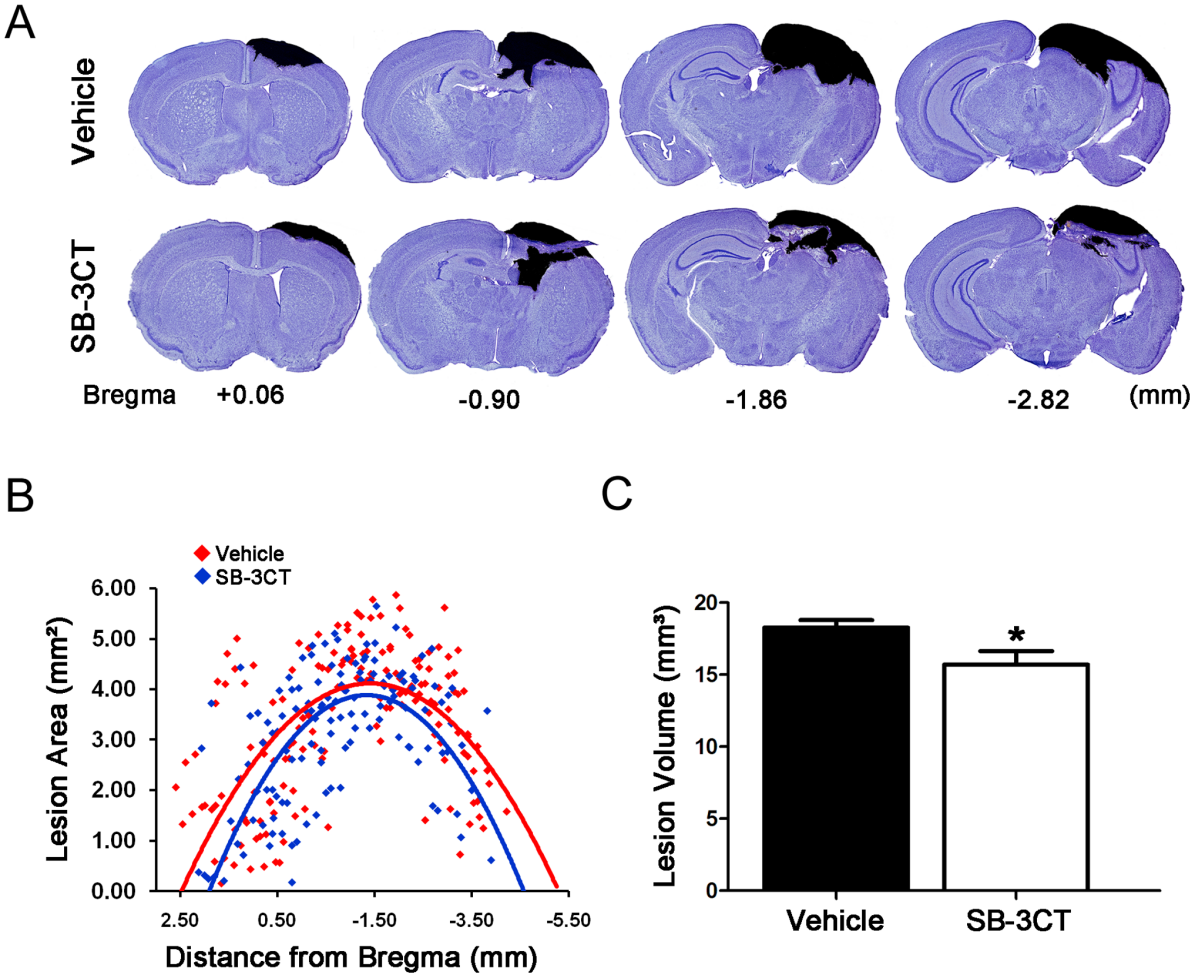
Histopathological quantification of lesion volumes in cresyl violet-stained brain sections at 7 days post-trauma. ***A.*** Representative cresyl violet-stained coronal brain sections from vehicle and SB-3CT-treated mice marked with their coordinates to Bregma. The black area in each section shows the contralateral hemisphere superimposed on top of the lesioned hemisphere to visualize the brain damage regions. ***B.*** Stereological scatter-plot of lesion areas in the cresyl-violet stained sections of vehicle and SB-3CT-treated mice at 7 days post-trauma. Each data point represents the lesion area in one cresyl violet-stained brain section, and plotted according to the rostro-caudal axis of the brain coordinate to Bregma. A second degree polynomial was generated to fit data points to visualize data trends. The graphs indicate a difference in lesion area between vehicle and SB-3CT-treated mice. ***C.*** Quantification of cortical lesion volume at 7 days post-trauma in the SB-3CT-treated mice compared to the vehicle-treated mice. n = 6 in each group; *, p<0.05 by one-tailed, unpaired Student’s *t*-test. Data expressed as mean ± SEM.

To further evaluate the effects of SB-3CT treatment on brain injury, we conducted histological assessment of lesion volume using the digital pathology whole slide imaging (WSI) technique combined with the unbiased stereology analysis. Over 25 serial brain sections in 40-µm thickness with the precise 200-µm interval were obtained from both the SB-3CT- and vehicle-treated mice 7 days after TBI. All brains showed severe contusion injuries with substantial tissue loss in the cortex, as well as damage in the underlying hippocampal region (Figure 4A). SB-3CT treatment for 7 days resulted in a significant reduction (Figures 4B) in cortical lesion volume - the SB-3CT-treated animals (15. 71±0.93 mm^3^) compared to the vehicle-treated mice (18.28±0.51 mm^3^) (Figures 4C) indicating an approximate 14 percent reduction in brain damage after treatment with SB-3CT.

### SB-3CT Treatment Protects Neurons from Dendritic Degeneration

**Figure 5:**
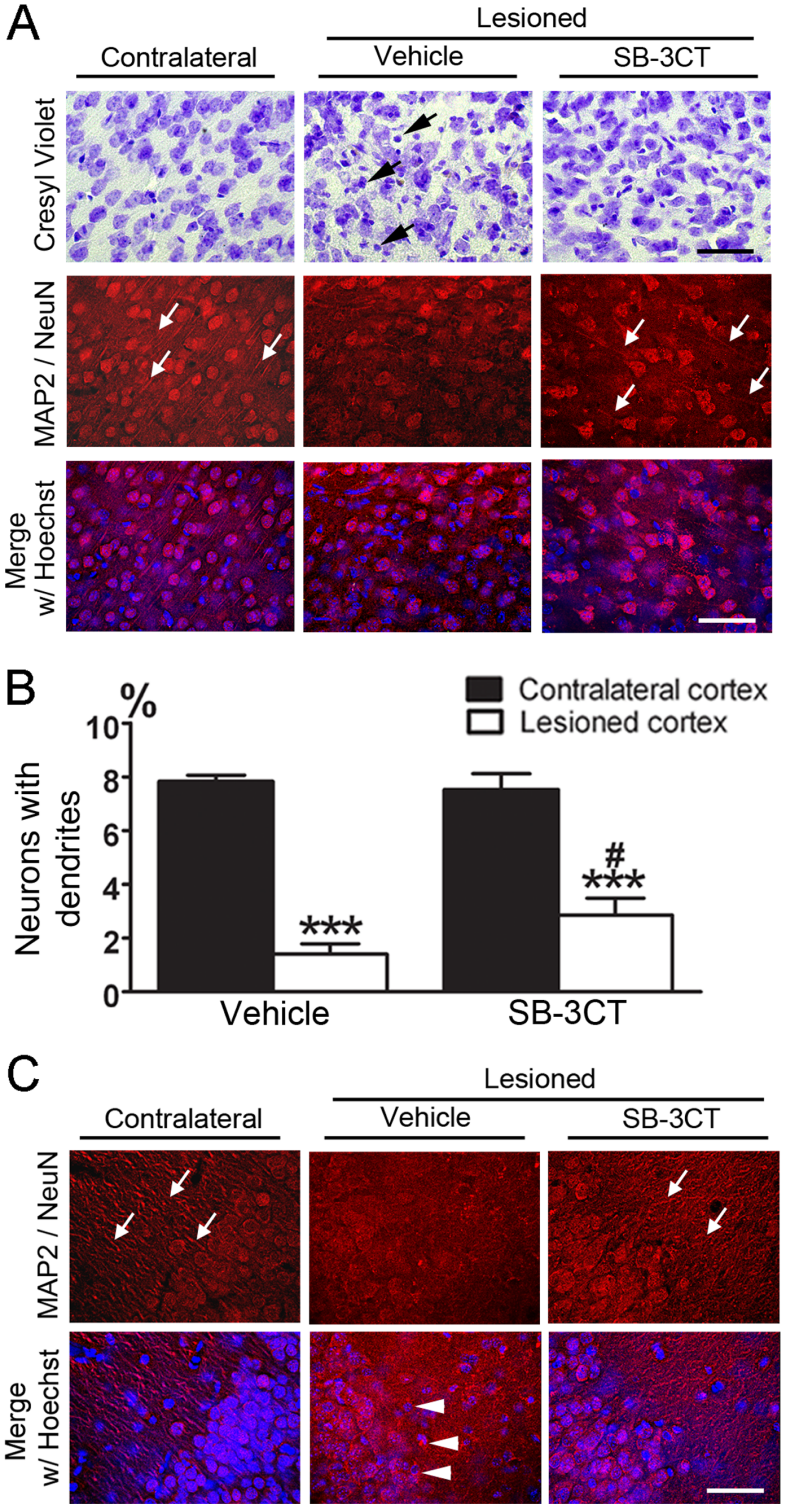
SB-3CT treatment protects cortical and hippocampal neurons from dendritic degeneration at 7 days after TBI. ***A.*** Representative photomicrographs of mouse cortical region in sections stained with either cresyl violet (top row), neuronal markers NeuN and MAP-2 (second row) and merged images with Hoechst dye counterstaining (bottom row) showing neuronal cell death and dendritic degeneration. SB-3CT treatment resulted in less irregular, darker stained neurons compared to the vehicle-treated mice (top row). In addition, more neurons with well-defined dendritic processes (white arrows) were seen in the contralateral cortex compared to the lesioned ones (second row). Scale bar (both black and white) = 50 µm. ***B.*** Quantification of neuronal cells with dendrites. Numbers of neurons with dendrites were counted from a total of approximately 800–1000 cells in each hemisphere. A marked difference in the percentage of neurons with dendrites was seen between contralateral and lesioned cortex. The percentage of neurons with dendrites was significantly higher in the lesioned cortex of SB-3CT-treated mice compared to that of vehicle-treated mice; n = 5 for each group; ***, p<0.001, comparing the lesioned to contralateral cortex; ^#^, p<0.05, comparing the difference between the contralateral and lesioned cortex after SB-3CT treatment to that in vehicle-treated animals using a one-tailed, unpaired Student’s *t*-test. Data are expressed as mean ± SEM. ***C.*** Comparison of dendritic degeneration in the lesioned and contralateral CA3 subregion of the hippocampus. Neuronal cells in the lesioned CA3 appear in condensed, irregular shape (white arrowheads), while cell bodies in the contralateral region as well as after SB-3CT-treatment appear intact in round shape with dendritic processes (white arrows), indicating that SB-3CT protects against dendritic degeneration from traumatic insult. Scale bar = 50 µm.

Excessive MMP-9 activity is known to enhance neuronal apoptosis and degeneration of neurovascular units in the CNS. In the present study, immunohistochemistry was conducted using the neuronal markers MAP2 and NeuN, and histochemical cresyl violet staining was carried out to visualize dendritic processes and neuronal cell bodies 7 days after TBI, in the presence or absence of SB-3CT treatment. Cresyl violet staining revealed substantial damage in neuronal morphology with irregular, condensed cell bodies (Figure 5A top row, indicated by black arrows) in the lesioned cortex compared to the corresponding contralateral region, which showed round, pale-stained healthy neurons. SB-3CT treatment preserved the morphology of neurons (Figure 5A top row) in the CCI-induced lesion regions. Immunohistochemistry of neuronal markers, NeuN and MAP2, showed that more neurons with well-defined dendritic processes (white arrows) were seen in the contralateral cortex compared to the lesioned region (Figure 5A second row). The number of surviving neurons with dendrites in the lesioned cortex was significantly higher in SB-3CT-treated mice (2.86±0.63%) than in vehicle-treated mice (1.40±0.39%, Figure 5B), while they were comparable in the contralateral cortex (7.53±0.59% and 7.84±0.23% with and without SB-3CT, respectively). Additionally, in the absence of SB-3CT, dendritic processes in the CA3 subregion of the lesioned hippocampus also exhibited remarkable damage with loss of the majority of dendrites, as well as neuronal cell death compared to the contralateral hippocampus (Figure 5C). These data suggested that inhibition of MMP-9 activity decreases the degeneration of neuronal dendrites and loss of cell bodies in both the cortex and hippocampus of the lesioned hemisphere following TBI.

### SB-3CT Treatment Attenuates Microglial Activation and Astrogliosis

**Figure 6:**
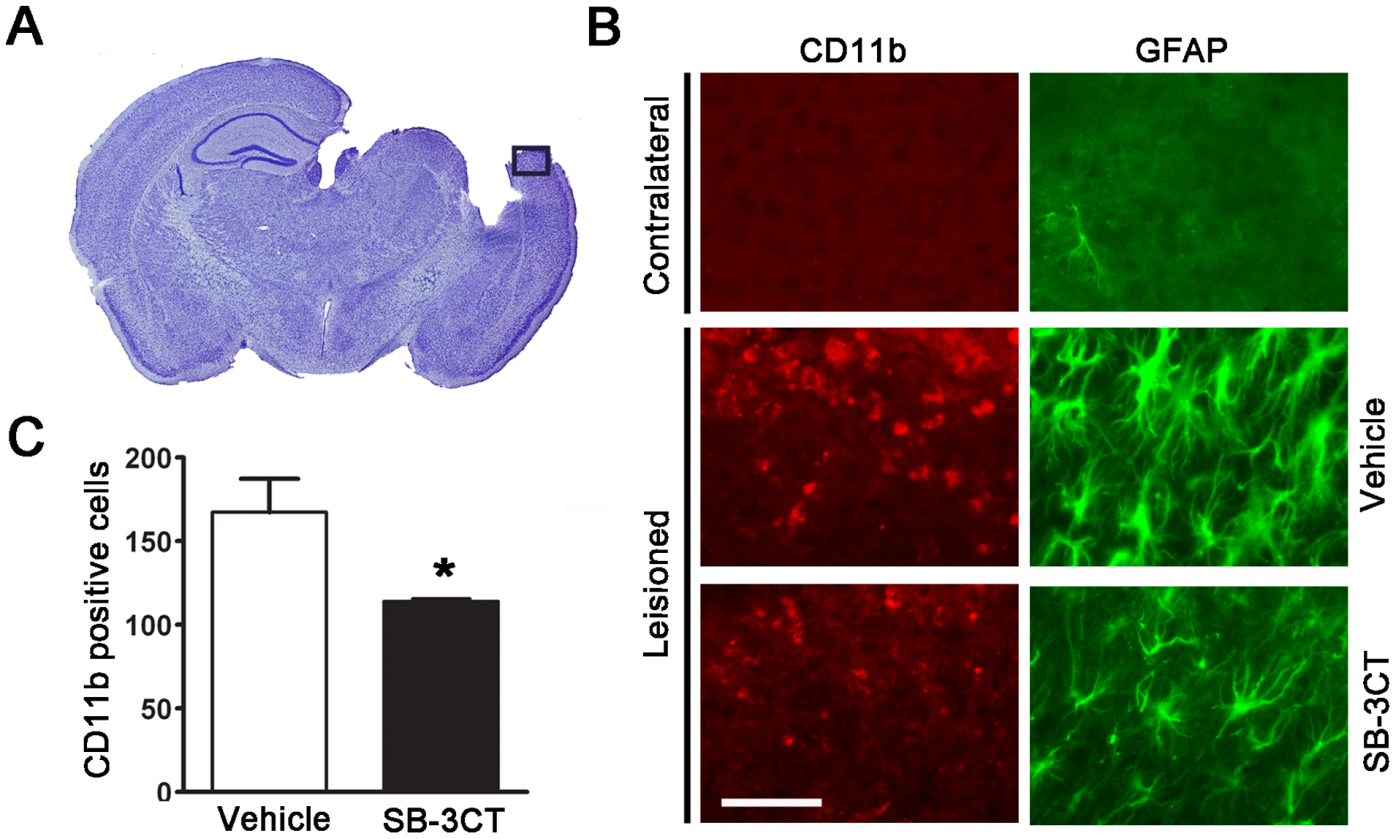
SB-3CT suppressed microglia activation and astrogliosis 7 days post-trauma. ***A.*** A representative microphotograph showing the cortical area for glial cell activation analysis. ***B.*** photomicrographs of cortical region in coronal sections of mouse brain immunofluorescently stained with microglial marker CD11b and astrocytic marker GFAP showing increased astroglial activity 7 days post-trauma. SB-3CT treatment attenuated activation of microglia and astrocytes in the lesioned cortex. Scale bar = 50 µm. ***C.*** Numbers of the CD11b positive microglia were counted from a total of approximately 1600 cells in the area showed in ***A*** **.** The number of CD11b positive microglia was significantly lower in the lesioned cortex of SB-3CT-treated mice compared to vehicle-treated animals; n = 3 for each group; *, p<0.05 by one-tailed, unpaired Student’s *t*-test. Data are expressed as mean ± SEM.

Neuroinflammatory responses including astrogliosis and microglial activation often accompany brain damage [10], [27]–[30]. We, therefore, examined microglia and astrocytes in the mouse brain after TBI by immunofluorescent staining with microglial marker CD11b and astrocytic marker GFAP. As shown in Figure 6, increased immunoreactivity of CD11b-positive microglia was observed in the lesioned cortex at 7 days post-trauma (Figure 6B), and seen as early as 24 hours after TBI (data not shown). The amoeboid form of activated microglia [31] was observed in the lesioned cortex, but not in the contralateral cortex. SB-3CT treatment for 7 days attenuated the number of amoeboid microglia. The numbers of CD11b-positive microglial cells were significantly reduced in the lesioned cortex of SB-3CT-treated mice at 7 days post-trauma compared to the vehicle-treated animals (Figure 6C). Additionally, reactive astrocytes with increased cytoplasm around the nucleus were found in the lesioned cortex, while fewer non-reactive astrocytes were observed in the contralateral cortex. SB-3CT treatment partially attenuated immunoreactivity of the GFAP-stained astrocytes (Figure 6B). Moreover, cresyl violet staining showed damage in the cortex and the hippocampal CA1 region, which was further validated by activation of microglial cells with isolectin-B4 histochemical staining (Figure S1). These results are consistent with previous observations showing microglial activation and astrogliosis after TBI [29], [32].

### SB-3CT Provides Long-term Protection from Cortical Damage and Sensorimotor Deficits after TBI

**Figure 7:**
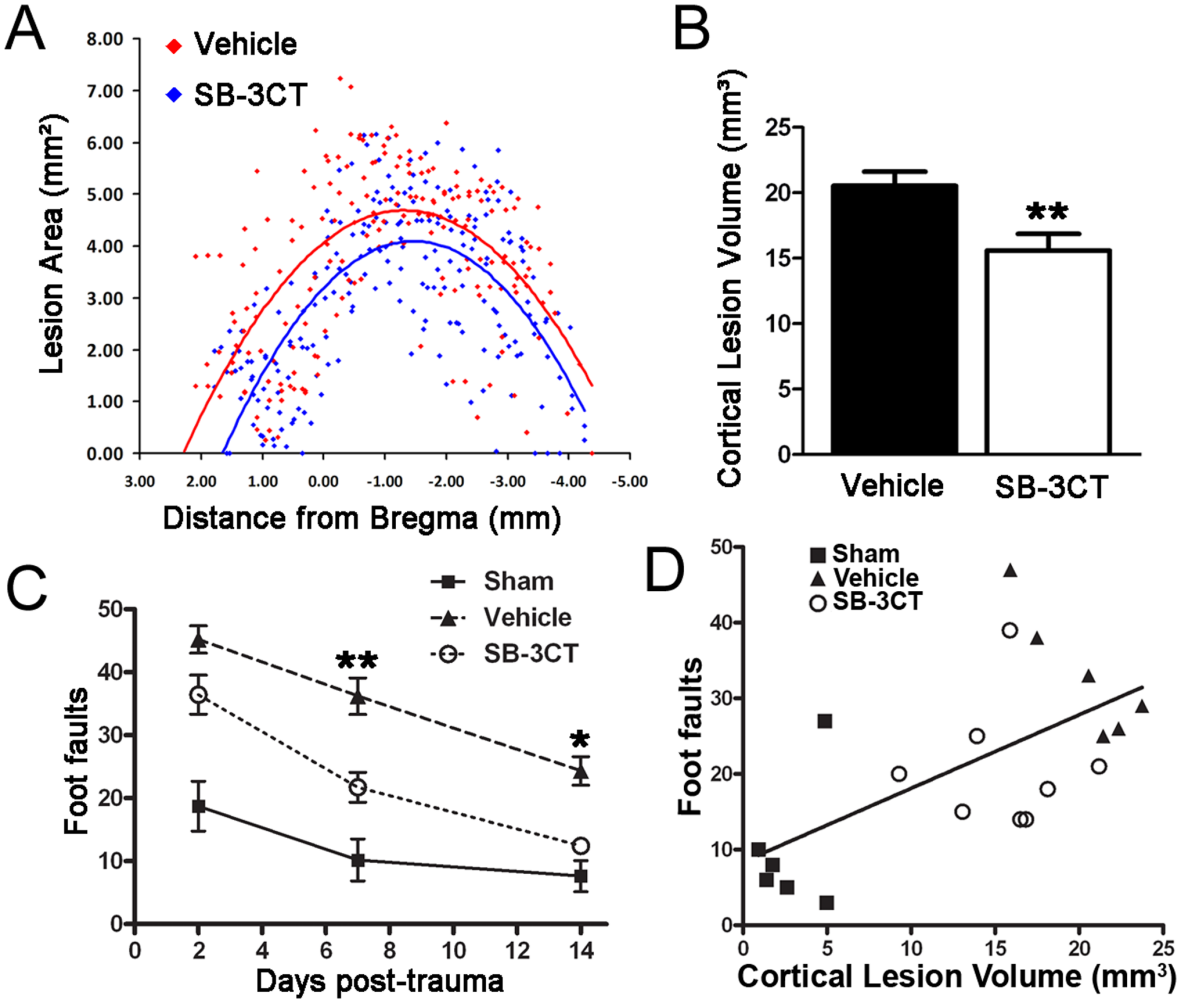
Protective effect of SB-3CT against long-term cortical damage and motor deficits after TBI. ***A.*** Histopathological quantification of cortical lesion area in cresyl-violet stained brain sections from vehicle and SB-3CT-treated mice at 30 days post-trauma. Cortical lesion areas were markedly smaller in SB-3CT-treated mice compared to the vehicle-treated mice. ***B.*** Quantification of cortical lesion volume in vehicle and SB-3CT-treated mice at 30 days post-trauma. **, p<0.01 by one-tailed, unpaired Student’s *t*-test; n = 7 in vehicle-treated, 8 in SB-3CT-treated mice. Data are expressed as mean ± SEM. ***C.*** Beam-walking test. SB-3CT-treated mice committed significantly fewer foot faults compared to vehicle-treated animals on days 7 and 14; *, p<0.05, **, p<0.01; but no significant difference between SB-3CT-treated and sham groups, by two-way repeated-measures ANOVA, Bonferroni post tests; n = 11 in sham, 10 in vehicle-treated, and 11 in SB-3CT-treated mice. Data are expressed as mean ± SEM. ***D.*** Correlation between cortical damage and motor deficits. Cortical damage areas between 7 days and 30 days after TBI were not significantly different. Cortical lesion area at 30 days and beam-walking foot faults at 7 days were correlated by one-tailed Pearson correlation test, Pearson r = 0.6192, p<0.01; n = 6 in sham, 6 in vehicle-treated, and 8 in SB-3CT-treated mice, showing that the correlation is significant. These three groups–sham, vehicle-treated and SB-3CT-treated mice, display good separation.

To assess the long-term effects of SB-3CT treatment, we assessed the histopathological differences 30 days post-trauma by analysis of cresyl violet-stained serial sections. A second degree polynomial was generated to fit the data points for each treatment group (vehicle and SB-3CT) and to visualize data trends (Figure 7A). This indicates a difference in lesion area between vehicle and SB-3CT-treated mice. We found that SB-3CT-treated animals exhibited cortical lesion volumes that were significantly smaller by approximately 24 percent than those of vehicle-treated animals (cortical lesion volume of 20.54±1.07 mm^3^ in vehicle-treated mice and 15.60±1.26 mm^3^ in SB-3CT-treated mice) (Figure 7B). There was no apparent cell damage in the brain sections of the mice that underwent sham operation (data not shown).

To evaluate long-term neurological function, we conducted behavioral assessments of the TBI mice treated with SB-3CT or vehicle. We used a beam-walking task in order to test fine motor coordination during the first 14 days post-trauma (Figure 7C). A two-way repeated-measures ANOVA analysis revealed a significant interaction (p = 0.019), as well as significant main effects of days (p<0.0001) and groups (p<0.0001), indicating that treatment with SB-3CT accelerates motor function recovery after TBI. SB-3CT reduced motor deficits by more than 50 percent compared to the vehicle-treated mice on day 7 after TBI, and resulted in an almost-complete recovery on day 14 (*, p<0.05 vehicle vs. sham or SB-3CT, Bonferroni post-tests). Moreover, quantitative data of the 30-day cortical lesion volume (Figure 7B) and 7-day beam-walking foot-faults (Figure 7C) were well correlated (Pearson r = 0.6192, p<0.01) (Figure 7D).

### SB-3CT Provides Long-term Protection from Hippocampal Damage and Cognitive Deficits after TBI

**Figure 8:**
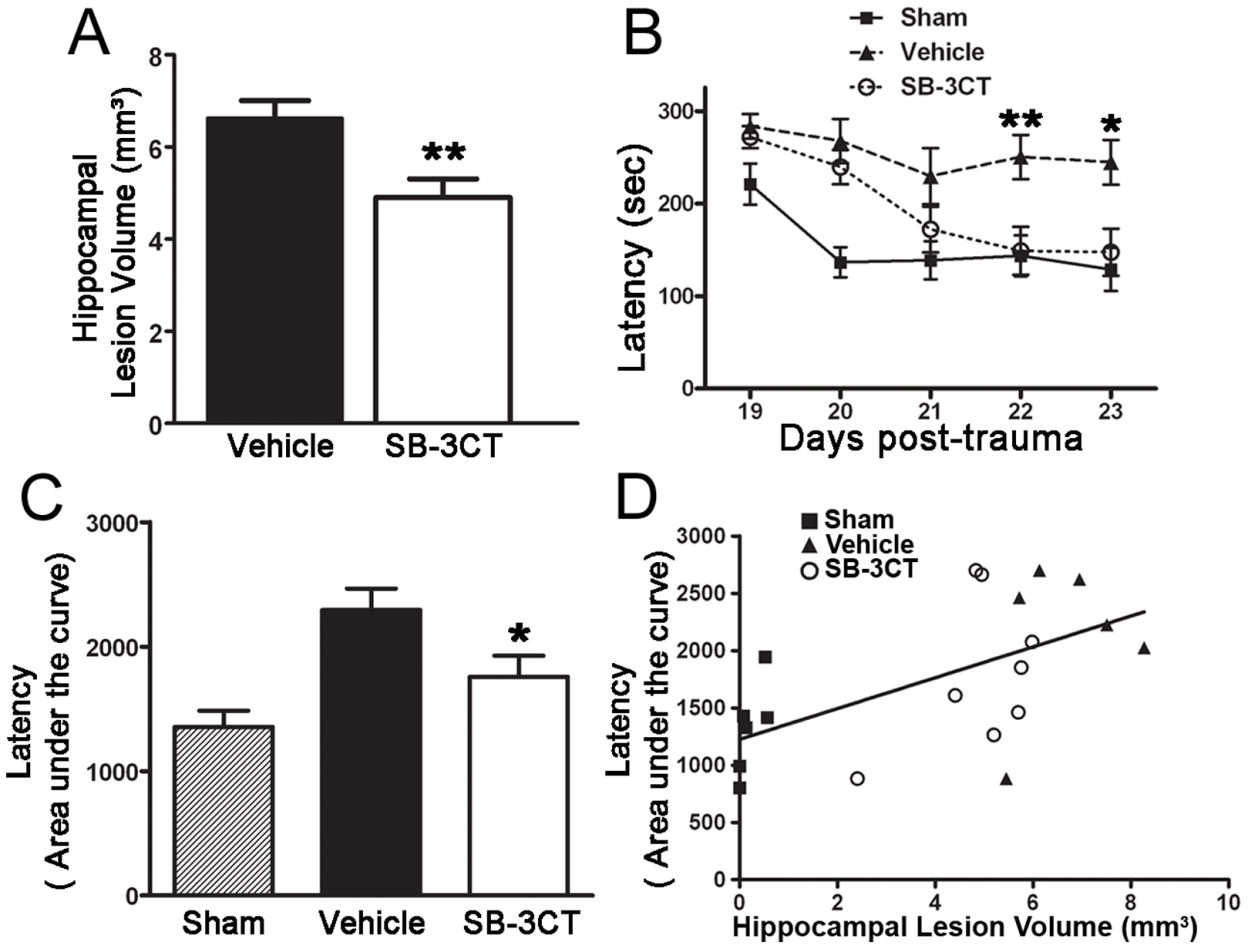
Protective effect of SB-3CT against long-term hippocampal damage and cognitive deficits after TBI. ***A.*** Quantification of hippocampal lesion volume in vehicle and SB-3CT-treated mice, 30 days post-trauma. **, p<0.01 by one-tailed, unpaired Student’s t-test; n = 7 in vehicle-treated, 8 in SB-3CT-treated mice. Data are expressed as means ± SEM. ***B.*** Barnes maze acquisition. Testing consisted of 10 trials (2 trials/day) over 5 days. Latency (sec) of each trial was measured. Two-way repeated-measures ANOVA revealed a significant interaction (p = 0.0149) and significant main effects of days (p<0.0001) and groups (p = 0.0011). SB-3CT treated mice performed significantly better than vehicle-treated mice on days 22 and 23 after TBI; *, p<0.05, **, p<0.01; n = 11 in sham, 10 in vehicle-treated, and 11 in SB-3CT-treated mice. Data are expressed as means ± SEM. ***C.*** For analysis of memory acquisition in the maze, the latency AUC over 10 trials was calculated for each animal, and group comparisons were performed by one-way ANOVA, Dunnett’s post test, showing that SB-3CT ameliorates cognitive deficits after TBI; *, p<0.05 vs. vehicle-treated; n = 11 in sham, 10 in vehicle-treated, and 11 in SB-3CT-treated mice. Data are expressed as means ± SEM. ***D.*** Correlation between hippocampal damage and memory deficits. Hippocampal lesion volumes in 30 days and AUC were correlated by one-tailed Pearson correlation test. Pearson r = 0.5817, p<0.01; n = 6 in sham, 6 in vehicle-treated, and 8 in SB-3CT-treated mice. These data indicated significant correlation and three groups: sham, vehicle-treated, and SB-3CT treated showed good separation.

Spatial learning requires memory processing in the hippocampus. We therefore examined the effects of SB-3CT on the ability to reduce hippocampal lesion volumes and ameliorate spatial learning and memory deficits due to TBI. We observed that SB-3CT-treated mice had hippocampal lesion volumes that were significant smaller by approximately 26 percent than those of vehicle-treated mice (hippocampal lesion volume of 6.62±0.38 mm^3^ in vehicle-treated mice and 4.91±0.40 mm^3^ in SB-3CT-treated mice) (Figure 8A). Spatial learning and memory were evaluated using a 5-day Barnes maze task, started at 18 days post-trauma. Measurement of latency revealed a significant impairment in spatial learning after TBI (Figure 8B). Similar findings were observed by measurement of errors (Figure S2). Two-way repeated-measures ANOVA showed a significant interaction (p = 0.0149 for latency, and p<0.0001 for errors) and significant main effects of days (p<0.0001 for both) and groups (p = 0.0011 for latency and p = 0.0029 for errors). Despite different levels of performance at the beginning of the Barnes maze, the sham-operated and SB-3CT-treated mice performed comparably to each other on the last three days of acquisition (p>0.05). The performance of the vehicle-treated mice were significantly worse compared to the sham-operated mice, while SB-3CT treatment significantly attenuated both latency and error numbers on the last two days of the trials (**, p<0.01 and *, p<0.05 vehicle vs. sham or SB-3CT, Bonferroni post-tests). For analysis of memory acquisition in the maze, the latency area under the curve (AUC) from trial one to trial ten (5 days) was calculated for each animal (Figure 8C). Group comparisons performed by one-way ANOVA revealed that SB-3CT treatment ameliorated cognitive deficits after TBI (*, p<0.05 vs. vehicle). Moreover, quantitative data for the 30-day hippocampal lesion volume (Figure 8A) and latency AUC (Figure 8C) show significant correlation (Pearson r = 0.5817, p<0.01), and indicated good separation on brain lesion and neurobehavioral deficits among the three groups: sham, vehicle-treated, and SB-3CT treated mice (Figure 7D and Figure 8D).

## Discussion

Biochemical events following the primary injury after TBI are complex depending on the extent of brain damage. In most instances, secondary injury is triggered by onset of a cascade of events that damages the BBB and is followed by inflammation, neurotoxicity, and edema in the brain. While brain damage due to the primary insult is difficult to avoid, the ensuing complications resulting from the secondary injury could, in principle, be mitigated by pharmacological intervention. Thus, identification of the factors responsible for secondary injury provides opportunities for therapeutic intervention. Substantial evidence indicates that MMPs, particularly MMP-9, play a critical role in degrading the ECM leading to anoikis–a form of cell death by losing the cell-cell interaction [8]. In addition, excessive MMP-9 activity can exacerbate pathological outcomes such as disruption of BBB [3], and enhances neuronal apoptosis and degeneration of neurovascular units after trauma and stroke [9], [15], [16], [33]–[35]. On the other hand, other MMPs can provide protective functions, such as those in neurovascular remodeling and microvascular recanalization [20], [36]. Thus, selective inhibition of MMP-9 would ameliorate secondary injury, while leaving the beneficial effect of other MMPs unaffected.

In the present study, we observed a dramatic increase in levels of proMMP-9 and activated MMP-9 in the lesioned cortex within 24 hours after TBI. Although the activated MMP-9 levels decreased rapidly after 1 day, the proMMP-9 levels in the lesioned cortex remained elevated for 10 days after TBI (Figure 1A). Interestingly, we also noted slight increases in proMMP-9 in the contralateral cortex at 24 hours post-trauma (Figure 1), probably due to the effect of the 15° angled, trans-hemispheric CCI induction causing a countrecoup injury. A previous report indicated that proMMP-9 levels are elevated as early as 3 hours after trauma and remain elevated for an entire week [22]. Despite the changes in MMP-9 levels, our results show no significant changes in MMP-2 levels at different time points within two weeks after TBI. These results are consistent with previous reports on human cerebrospinal fluid samples obtained after trauma [12], on traumatic post-mortem tissues [37], and in other neurological disease models [9], [16].

In this study, we evaluated the selective mechanism-based MMP-9 inhibitor, SB-3CT, for its ability to attenuate brain damage in a severe TBI model in mice induced by the EM impactor. Two features are worth mentioning about this inhibitor. First, as documented by the pharmacokinetic experiments in the present report, SB-3CT can cross the BBB in mice within minutes of administration (Figure 3) and achieves therapeutic concentrations in the brain. The unique pharmacokinetic properties of SB-3CT indicate that this compound could be used for early intervention after TBI. Secondly, SB-3CT is a selective gelatinase inhibitor that works only after binding to the active site of the enzymes, and thus allowing a slow-binding inhibition, which does not readily reverses [38]. As such, SB-3CT affords unparalleled selectivity in targeting gelatinases as demonstrated in various neurological diseases [9], [15], [16], [33], [39].

SB-3CT may contribute to the therapeutic treatment of TBI through several mechanisms. This compound seems to specifically interact with active MMP-9, as well as reduce proMMP-9 expression levels (Figure 2). Although the mechanism for the latter action is still not well understood, there is evidence that it may involve a feedback loop that controls expression of the protein via a transcription mechanism, such as nuclear factor-κB (NF-κB) and AP-1 [36]. A recent report detailed a signaling cascade in which secretion of apolipoprotein E from astrocytes activates the proinflammatory cyclophilin A-NF-κB pathway, resulting in MMP-9 induction [40]. In this regard, SB-3CT may inhibit active MMP-9 through the positive feedback loop that regulates the induction of proMMP-9 expression. Our earlier study demonstrated the effect of SB-3CT on attenuating ECM proteolysis after ischemic stroke in mice [15]. Other studies have demonstrated that SB-3CT can ameliorate ECM degradation, thus reducing BBB disruption and edema [9], [39], [41].

Results from this study demonstrate the ability of SB-3CT to protect neurons from dendritic degeneration, as well as attenuate astrogliosis and microglial activation (Figures 5 and 6). It is likely that this pleiotropic effect of SB-3CT arises at its specific target MMP-9. Most significantly, treatment with SB-3CT for 7 days could prevent spreading of brain damage, which was observable even after 30 days post-trauma. The protective effects were supported by its ability to ameliorate long-term sensorimotor and cognitive deficits after TBI, suggesting the importance of using this highly selective inhibitor to target excessive activity of specific MMPs and mitigate the detrimental effects during the secondary injury phase of TBI.

Previous studies demonstrated that SB-3CT was metabolized to *p*-OH SB-3CT by hydroxylation of the terminal phenyl ring at the *para*-position as the major metabolic pathway [17], [18]. In this study, we found that SB-3CT and its active metabolite *p*-OH SB-3CT can cross the BBB and enter the brain parenchyma to exert inhibitory effects (Figure 3 and Table 1). In the paradigm in which SB-3CT was given in multiple doses, brain levels of SB-3CT were above the *K_i_* for MMP-9 (400 nM) at 30 minutes after the last dose. As SB-3CT is a slow-binding inhibitor, the reversal of inhibition occurs very slowly. Thus, the levels of SB-3CT observed in the brain result in effective inhibition of MMP-9. On the other hand, brain levels of *p*-OH SB-3CT were below the *K_i_* for MMP-9 of 160 nM at the time points examined (Table 1). Given that brain *AUC*_0–∞_ for SB-3CT is 19-fold higher than that for *p*-OH SB-3CT (87.8 pmole·minutes/mg vs. 4.61 pmole·minutes/mg), it is reasonable to conclude that the efficacy observed in the TBI model (see Figure 4) is likely due to SB-3CT rather than to its active metabolite *p*-OH SB-3CT. Nevertheless, both SB-3CT and *p*-OH SB-3CT are distributed rapidly to all regions of the brain (Table 2), with maximal levels observed at 10 minutes (the first time point collected). SB-3CT and *p*-OH SB-3CT do not accumulate in the brain even after repeated-dose administration, with half-lives of elimination of 53 and 187 minutes, respectively. Since more than 98% of small-molecule drugs do not cross the BBB [13], [42], our results conclusively demonstrate the ability of SB-3CT to be rapidly absorbed and distributed in the brain. These desirable pharmacokinetic properties, together with its lack of accumulation in the brain, make this class of compounds highly promising for the treatment of acute neurological diseases.

Besides protection of neurons from degeneration and glial cells from activation (Figure 5 and 6), the present study further showed the effect of SB-3CT in rescuing brain from prolonged damage by approximately 24 to 26 percent reduction in lesion volumes of the cortical and hippocampal subregions (Figure 7 and 8). More interestingly, there was a sustained effect of SB-3CT treatment, as we observed profound amelioration of fine motor coordination related to cortical lesion and hippocampus-associated spatial learning and memory. These results are important in explaining the improved neurobehavioral outcomes in sensorimotor and cognitive function in mice subjected to TBI.

Taken together, this study shows that MMP-9 is an important target for therapy to attenuate secondary injury cascades and that SB-3CT mitigates the detrimental effects of MMP-9 over-activation. Thus, the SB-3CT class of mechanism-based gelatinase inhibitors is a promising therapy to attenuate secondary injury after TBI.

## Materials and Methods

### TBI Induction

All animal procedures were performed under the compliance with protocol approval by the University of Missouri Animal Care and Use Committee, which is in accordance with the National Institutes of Health Guide for the Care and Use of Laboratory Animals. The TBI procedure employed an EM impactor for CCI and was conducted as previously described [21]. A total of 86 mice were used in this study. Briefly, 8–10 week-old adult male C57BL/6J mice (The Jackson Laboratory, Bar Harbor, ME) weighing 20–25 g were anesthetized with 2.0% gaseous isoflurane in a nitrogen/oxygen mixture inside a sealed anesthesia chamber. Each mouse was then stably placed on a Kopf stereotaxic apparatus (David Kopf Instruments, Tujunga, CA). Body temperature was monitored with a rectal thermistor probe (TH-10Kmp, Cell MicroControls, Norfolk, VA), and maintained at a constant 37°C on a silicon heating pad (HS-3×2.5 Heater, Cell MicroControls, Norfolk, VA). Following a midline skin incision and removal of connective tissue under sterile conditions, a 5.0 mm diameter craniotomy was performed in the left parietotemporal skull using a pedal-operated high-speed micro-drill mounted on the stereotaxic arm. A 5.0-mm diameter bone disc was then removed to expose the left cortex, while keeping the dura mater intact. A MATLAB-controlled EM impactor (MyNeuroLab Stereotaxic Impactor, St. Louis, MO) with a 3.0-mm diameter tip was centered at 2.7 mm to the left of the midline suture and 3.0 mm rostral to lambda, at an angle of 15° with the vertical. Once the position was set, the EM impactor delivered a CCI with a velocity of 5.0 m/s and dwell time of 100 ms, at a depth of 2.5 mm. This operation produces a moderately severe contusion in the left parietotemporal cortex and the underlying hippocampus as marked by pronounced behavioral deficits, but virtually no mortality. Following impact, the original skull disc was placed back over the exposed cortex, and the incision was sutured. Each mouse was released from anesthesia and placed in an empty cage over a heating pad for recovery. All efforts were made to minimize animal suffering. For sham control mice used in the study, only the craniotomy was performed. To investigate the efficacy of SB-3CT, mice were injected i.p. with SB-3CT (12.5 mg/mL in 25% DMSO/65% PEG-200/10% water, 25 mg/kg body weight) or vehicle (25% DMSO/65% PEG-200/10% water) at 2 and 4 hours post-trauma, followed by one dose daily for the next 6 days. SB-3CT was synthesized as described previously [14]. Mice were observed daily for the duration of the study (7 or 30 days). For design of the 30-day long term study, animals in each group were double-blinded and randomly assigned into two different tissue process procedures after behavioral testing–mouse brains were either fixed and sectioned for histopathological evaluation, or freshly dissected and then frozen at −80°C for later analysis, reflecting the difference of the animal numbers mentioned in the legends of Figures 7 and 8.

### Gelatin Zymography

Gelatinase activity in brain homogenates was determined by gelatin zymography as described previously [15], [16]. Briefly, brains were quickly dissected into left and right cortical regions and stored immediately at −80°C until later analysis. Gelatinases were extracted in 10 mM Tris-buffered saline (TBS), pH 7.6, containing 5 mM CaCl_2_, 150 mM NaCl, 0.05% Brij 35, 0.02% NaN_3_, 1% Triton X-100, 100 µM PMSF, and a proteinase inhibitor cocktail (PIC; Sigma-Aldrich, St. Louis, MO) at 1∶100 dilution; and followed by affinity precipitation with gelatin-Sepharose 4B (GE Healthcare Bio-Sciences, Piscataway, NJ). Bound material was released from the beads using 10% DMSO in TBS, and samples were analyzed by electrophoresis in a 10% SDS-PAGE gel containing 0.1% gelatin under non-reducing conditions, followed by incubation overnight at 37°C in 50 mM Tris buffer, pH 8, with 5 mM CaCl_2,_ 200 mM NaCl, and 0.02% Brij 35. Gelatin-Sepharose 4B-enriched HT1080 cell conditioned media containing both MMP-2 and −9 or the purified recombinant MMP-9 (EMD Millipore, Billerica, MA) were used as positive controls. Gels were stained with Coomassie blue and digitized using a Perfection V750 PRO scanner (EPSON, Long Beach, CA). Densitometry was performed using ImageJ software by measuring the mean intensity of each gelatinolytic band in images of the gels and subtracting the mean intensity of the background measured in the area immediately below the band.

### Determination of SB-3CT and *p*-OH SB-3CT Levels in Plasma and Brain

The pharmacokinetics of SB-3CT and its metabolite *p*-OH SB-3CT, synthesized as previously described [14], [43], were investigated. Male C57Bl/6J mice (6–8 weeks old, 20–25 g body weight, specific pathogen free, The Jackson Laboratory, Bar Harbor, ME) were given 50 µL i.p. injections of a 10 mg/mL solution of SB-3CT in 25% DMSO/65% PEG-200/10% water (equivalent to 25 mg/kg) at 0, 2, and 24 hours (n = 3 per time point). Under anesthesia, terminal blood was collected in heparin tubes through the posterior vena cava at various time points after the last dose, and tubes were centrifuged to collect plasma. Whole brain samples were harvested after transcardiac perfusion with saline and immediately flash frozen in liquid nitrogen and stored at −80°C until analysis. For the study to examine regional brain distribution, mice (n = 9) were administered 25 mg/kg i.p. doses of SB-3CT at 0, 2, and 24 hours. At 10 minutes after the last dose, whole brain samples were rapidly dissected into different regions (cortex, striatum, hippocampus, cerebellum and brain stem), and immediately frozen in dry ice.

A 75-µL aliquot of plasma was mixed with 150 µL of internal standard in acetonitrile. The sample was centrifuged at 10,000×*g* for 10 minutes. Brain samples were weighed and homogenized for 5 minutes in one volume equivalent of cold acetonitrile, containing an internal standard, using a bullet blender (Next Advance, Inc., Averill Park, NY). For regional brain distribution, the striatum, hippocampus, cerebellum and brain stem samples were pooled from 3 different mice for analysis.

The homogenates were centrifuged twice at 20,000×*g* for 20 minutes at 4°C. The supernatants from plasma and brain samples were collected and analyzed by reversed-phase UPLC/ESI in the negative mode with MRM of the transitions 305→168 for SB-3CT and 321→184 for *p*-OH SB-3CT. Standard curves of SB-3CT and *p*-OH SB-3CT were prepared by fortification of blank mouse plasma (and blank brain) with SB-3CT or *p*-OH SB-3CT at various concentrations. Quantification was performed using peak area ratios relative to the internal standard and linear regression parameters were calculated from the calibration curve standards prepared in blank mouse plasma (and blank brain). The internal standard used in all of the analyses was *N*-(4-(4-((thiiran-2-ylmethyl)sulfonyl)phenoxy)phenyl)methanesulfonamide [43], which was analyzed by MRM using the transition 398→261. The chromatographic, MRM conditions, and method validation were the same as previously described [17].

Pharmacokinetic parameters were calculated as follows. The area under the mean concentration-time curve up to the last quantifiable sampling time (*AUC*_0–last_) was calculated by the trapezoidal rule using the pharmacokinetic software PK Solutions (Version 2.0, Summit Research Services, Montrose, CO). The AUC-time curve from time zero to infinity (*AUC*_0–∞_), representing systemic exposure of a therapeutic agent in the body, was calculated as *AUC*_0–last_+(*C*_last_/*k*), where *C*_last_ is the concentration at the last quantifiable sampling time and *k* is the elimination rate constant. Half-lives (*t*_½α_ and *t*_½β_) were estimated from the linear segment of the initial or terminal portion of the concentration-time data by linear regression, where the slope of the line was the rate constant *k* and *t*_½_ = ln 2*/k*.

### Tissue Processing, Histochemical Staining and Quantitative Assessment of Brain Lesion

At 7 or 30 days after TBI, mice were sacrificed and brains were processed for histochemical staining and assessment of brain lesion volumes using the stereology technique as described [44], [45]. Briefly, mice were sacrificed at appropriate experimental time points and transcardially perfused with 4% paraformaldehyde in 100 mM phosphate buffer (PB) and brains were dissected. Coronal sections were serially cut through the brains at 40 µm thickness with a vibratome (VT1200S, Leica Microsystems, Inc., Bannockbum, IL), and 120–150 tissue sections from each brain were sequentially collected into 24-well plates. Every 5^th^ section was mounted on poly-L-lysine coated glass slides and stained with cresyl violet to quantify brain lesion volumes and examine neuronal cell death.

For the histochemical staining of microglia, fixed sections were processed with GSA isolectin-B4-HRP (Microglial cell marker from *Griffonia simplicifolia*, L5391; Sigma Chemical Co, St. Louis, MO). Sections were first incubated in a solution of 0.1% Triton- X-100 in PBS containing 0.1 mM of CaCl_2_, MgCl_2_, and MnCl_2_ for 15 minutes, and then washed with 0.3% hydrogen peroxide (H_2_O_2_) in PBS for 10 minutes to deplete endogenous peroxidase activity. After rinsing with 0.1% Triton-X-100 in PBS, sections were incubated in a solution of isolectin-B4, peroxidase conjugate (20 µg/mL in PBS containing 0.1% Triton-X-100, 0.1mM of CaCl_2_, MgCl_2_, and MnCl_2_) for 2 hours at room temperature. Three washes with 0.1% Triton-X-100 in PBS preceded the final reaction with DAB-H_2_O_2_ for 5 ∼ 8 minutes, to allow for color development. Sections were then washed in PBS mounted on slides, and viewed with bright field microscopy.

Systematic evaluation of a large number of histological specimens in an unbiased manner requires high-speed imaging tools for the supervised analysis of pathological assessment to reduce quantitative variations. In this study, we applied an innovative approach by using an automatic multi-focus plane, high-throughput digital pathology system (Aperio ScanScope CS digital scanner, Vista, CA) for WSI of the cresyl violet-stained brain sections. Using the web-based ImageScope software package, the digital photomicrographs were then analyzed in a double-blinded manner, allowing the results to be validated independently, as described [46]. In order to calculate lesion volumes, areas of the contralateral and the lesioned cortex were measured in photomicrographs of each coronal section to obtain values in mm^2^. The area of the lesioned cortex was subtracted from that of the contralateral cortex. Sections were then assigned a position along the rostro-caudal axis of the brain based on the anterior-posterior axis of the brain coordinate to Bregma and the difference plotted on the y-axis versus the anterior-posterior coordinates of the section on the x-axis. A second degree polynomial was generated in MS Excel to best fit the data points in order to visualize data trends. To calculate lesion volumes, the lesion area of each section was multiplied by a step size of 200 µm (the distance between adjacent sections), and these values summed together in order to yield the lesion volume (in mm^3^) for each brain.

### Fluorescence Immunohistochemistry

Fluorescence immunohistochemistry was carried out as described [15], [16], [19]. For each brain analyzed, coronal sections were chosen near the center of the craniotomy (Bregma −1.50 mm) and adjacent to a section stained with cresyl violet for comparison. Each section was placed in PBS for 1 hour prior to immunostaining with the following antibodies: Neuronal markers–MAP-2 (1∶200, M4403, Clone HM-2; Sigma Chemical Co, St. Louis, MO) and NeuN (1∶200, MAB377, Clone A60; Millipore-Chemicon, Temecula, CA); astrogliosis by astrocyte marker – GFAP (1∶500, G9269; Sigma Chemical Co, St. Louis, MO), and microglial marker – rat anti-CD11b polyclonal antibody (1∶400, 550274; BD Biosciences, San Jose, CA). Sections were then visualized with fluorophore-conjugated secondary antibodies (1∶300, goat anti-mouse IgG-Alexa488, A11001; goat anti-mouse IgG-Alexa594, A11005; and goat anti-rabbit IgG-Alexa488, A110034; and goat anti-rat IgG-Alexa594, A11007; Life Technologies/Invitrogen, San Diego, CA). Sections were counterstained in a solution of Hoechst dye 33342 (1∶1000; H-3570, Molecular Probes-Invitrogen, San Diego, CA). Fluorescence photomicrographs of the cortex and hippocampal CA3 regions were captured using a Leica DMI 6000B fully automated epifluorescence microscope (Leica Microsystems Inc., Buffalo Grove, IL) and analyzed with AF6000 applications for deconvolution imaging.

### Behavioral Analyses

#### Beam-walking

Evaluation trials were conducted at 2, 7, and 14 days post-TBI surgery to assess motor function, as described [47] with modifications. The apparatus consisted of a horizontal wooden beam, 6.0 mm wide, 90.0 cm long, and elevated to 48.0 cm above ground. Each mouse was placed on one end of the beam and allowed to walk across it towards the opposite end into a dark goal box. The performance of animals was video recorded, and the number of foot faults for the right hind limb was counted during the first 50 steps. Experiments and analyses were performed by observers blinded to individual treatment. A basal level of competence at this task (<5 foot faults per 50 steps) was established prior to TBI induction surgery.

#### Barnes Maze

Maze design and testing were conducted as previously described [48], starting at 18 days post-trauma and 3 days after the last beam-walking task, allowing mice to take time to rest. The apparatus consisted of a circular platform, 75-cm in diameter, elevated 56.5 cm above the floor with 20 holes (each 5 cm in diameter) evenly spaced around the perimeter, allowing mice to escape under stimulation with three 100-watt lights. The platform was surrounded by a black wall with four visual cues (a triangle, square, circle, and cross) inside the wall. Black fabric curtains covered the floor beneath the maze apparatus and hung 150 cm high from floor level to ensure that the mice were using the visual cues provided in the maze, instead of distal cues within the testing room. Each mouse was assigned an escape hole number and the escape box location remained constant for any individual mouse across test trials. Behavioral testing consisted of two shaping trials on day 18 post-trauma, followed by 10 evaluation trials (2 trials/day) over a period of five days with a 30 minutes inter-trial interval. Each day, the animals were transferred from their cage room to the testing room 30 minutes prior to the start of testing; a trial began by placing the mouse under a black starting box positioned in the center of the platform. After 60 seconds, the box was lifted and the mouse had a maximum of 5 minutes to find and enter the escape box. Latency (time it took for the mouse to find the escape box) and total errors (nose-pokes into non-escape holes) were recorded. If the mouse did not enter the escape box within 5 minutes, it was gently guided there by the experimenter’s hand. After 30 seconds, the mouse was removed from the escape box and returned to its home cage.

### Statistical Analysis

All experiments were performed in a randomized-blinded manner. Data are expressed as mean values ± SEM and were analyzed by unpaired one-tailed Student's *t*-test for any two-group comparisons and one-way ANOVA using Dunnett’s post test for multiple-group comparisons. These analyses were predicted to one direction of the pharmacological effect, as we and others have reported in testing the efficacy of the gelatinase inhibitor on pathology and enzymology in the paradigms of cerebral ischemia, intracranial hemorrhage and spinal cord injury. Behavioral results were analyzed by two-way repeated-measures ANOVA with *Trials/Days* as within-subjects factors and *Groups* as a between-subjects factor and Bonferroni multiple comparisons using Prism 5 software (GraphPad Software, La Jolla, CA). Differences were considered significant at *p*<0.05 for all analyses.

## Supporting Information

Figure S1 **CCI-induced primary damage and microglial cell activation.** Representative photomicrograph showing a cresyl violet-stained coronal section of a mouse brain, 24 hours after CCI-induced brain injury. Substantial damage can be seen in the lesioned compared to the contralateral cortex. ***Top:*** Enlarged areas of hippocampal CA1 region reveal apparent loss of neuronal cell bodies (arrows) and damage to surrounding neuronal dendrites (arrowheads) in the lesioned hemisphere. ***Bottom:*** Activated microglial cells (arrows) stained with BS isolectin-B4 in the dentate gyrus of the hippocampus 24 hours post-trauma in the lesioned (left) compared to contralateral (right) hemisphere.
(TIF)

Figure S2 **Long term effect of SB-3CT on spatial learning after TBI.** Barnes maze acquisition consisted of 10 trials (2 trials/day) over 5 days. Errors (numbers) by day were measured. Two-way repeated-measures ANOVA revealed a significant interaction (p<0.0001) and significant main effects of days (p<0.0001) and groups (p = 0.0029). SB-3CT-treated mice performed better than vehicle-treated mice on day 22 and 23 days after TBI *, p<0.05; n = 11 in sham, 10 in vehicle-treated, and 11 in SB-3CT-treated mice. Data are expressed as means ± SEM.
(TIF)
