# Supplementary figures and images for: Selective Inhibition of Matrix Metalloproteinase-9 Attenuates Secondary Damage Resulting from Severe Traumatic Brain Injury
Source: PLoS One. 2013 Oct 23;8(10):e76904. doi: 10.1371/journal.pone.0076904 (PMC3806745; doi:10.1371/journal.pone.0076904)

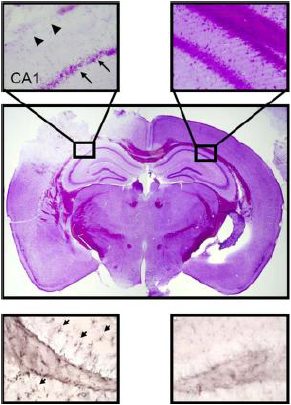

Supplement: Figure S1 — CCI-induced primary damage and microglial cell activation. Representative photomicrograph showing a cresyl violet-stained coronal section of a mouse brain, 24 hours after CCI-induced brain injury. Substantial damage can be seen in the lesioned compared to the contralateral cortex. Top: Enlarged areas of hippocampal CA1 region reveal apparent loss of neuronal cell bodies (arrows) and damage to surrounding neuronal dendrites (arrowheads) in the lesioned hemisphere. Bottom: Activated microglial cells (arrows) stained with BS isolectin-B4 in the dentate gyrus of the hippocampus 24 hours post-trauma in the lesioned (left) compared to contralateral (right) hemisphere. (TIF) [file pone.0076904.s001.tif]

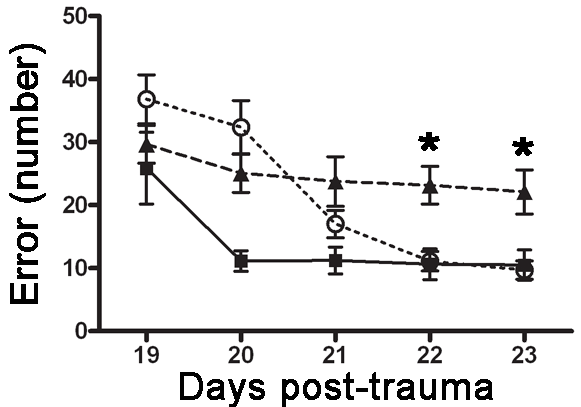

Supplement: Figure S2 — Long term effect of SB-3CT on spatial learning after TBI. Barnes maze acquisition consisted of 10 trials (2 trials/day) over 5 days. Errors (numbers) by day were measured. Two-way repeated-measures ANOVA revealed a significant interaction (p<0.0001) and significant main effects of days (p<0.0001) and groups (p = 0.0029). SB-3CT-treated mice performed better than vehicle-treated mice on day 22 and 23 days after TBI *, p<0.05; n = 11 in sham, 10 in vehicle-treated, and 11 in SB-3CT-treated mice. Data are expressed as means ± SEM. (TIF) [file pone.0076904.s002.tif]
